# Assessing metabolic health in a general population: A comparative analysis of three definitions in the Tromsø Study 2015–2016

**DOI:** 10.1371/journal.pone.0333402

**Published:** 2025-10-06

**Authors:** Monika Lund Machlik, Sameline Grimsgaard, Laila A. Hopstock, Bjarne K. Jacobsen, Marie W. Lundblad

**Affiliations:** 1 Department of Community Medicine, UiT The Arctic University of Norway, Tromsø, Norway; 2 Department of Health Care and Sciences, UiT The Arctic University of Norway, Tromsø, Norway; 3 Department of Community Medicine, Centre for Sami Health Research, UiT The Arctic University of Norway, Tromsø, Norway; Endocrinology and Metabolism Population Sciences Institute, Tehran University of Medical Sciences, IRAN, ISLAMIC REPUBLIC OF

## Abstract

**Background:**

The concept of metabolically healthy individuals with obesity (MHO) has gained considerable interest. Nevertheless, the lack of a standardized definition for metabolic health complicates the comparison of the prevalence and health implications of MHO.

**Aim:**

To compare three definitions of metabolic health in terms of their prevalence, overlap, and frequency with which criteria are met within a general population.

**Methods:**

We used data from 20 581 women and men aged 40–99 years attending the seventh survey of the Tromsø Study (Tromsø7) in 2015–2016. Participants were classified as metabolically healthy (MH) by definitions A) resembling metabolic syndrome (MetS) requiring ≤1/4 MetS components; B) strict requiring fulfillment of 0/4 MetS components, and C) empirically derived definition requiring fulfillment of 0/3 components including diabetes, elevated blood pressure and waist-to-hip ratio. Prevalence of MH was assessed descriptively in categories of normal weight, overweight and obesity based on body mass index (BMI). We used Venn diagrams to present the overlap between the three definitions applied to identify MH individuals, and the frequency of fulfilled components in metabolically unhealthy (MU) individuals (not classified as MH). All analyses were stratified by sex.

**Results:**

Prevalence of MH was higher in women and participants in lower BMI categories. Using definition A, 50% of women and 38% of men with obesity were classified as MH. Under definition B, 18% of women and 10% of men with obesity were considered MH. Definition C resulted in prevalences of 29% and 18%, in women and men with obesity, respectively. Blood pressure was the most common component in MU individuals, met by 76%−89% of MU women and 81%−93% of MU men, depending on the definition.

**Conclusion:**

The considerable variation in MH prevalence across different definitions underscores the need for a consensus definition, to further establish public prevention and clinical treatment strategies.

## Introduction

Metabolic risk factors, such as elevated levels of blood pressure, plasma triglycerides and glucose, waist circumference and low levels of high-density lipoprotein cholesterol (HDL-C), often coexist, and are associated to a higher risk of cardiovascular disease (CVD) and type 2 diabetes mellitus [1]. Individuals with such metabolic profiles can be considered metabolically unhealthy (MU), while those without can be considered metabolically healthy (MH). However, no standardized definition exists to discriminate between the MH and MU phenotypes [2,3]. Due to the close link between obesity and metabolic risk factors [4], individuals having obesity and simultaneously a healthy metabolic profile have been of particular interest to research. This phenomenon is called metabolically healthy obesity (MHO). However, 30 different definitions of MHO have been identified, resulting in prevalences of MHO ranging from 6–75% in population-based studies from Europe, USA, Asia, and Australia [3]. The large variety of definitions complicates the assessment of metabolic health status, the understanding of its health risks, and the implication of health initiatives aimed at preventing or treating metabolic-related diseases.

The most common approach to determine metabolic health status is to use the metabolic syndrome (MetS) components [5]: blood pressure, plasma HDL-C, triglycerides, and glucose [2,3,6]. The most widely used definition of MHO requires that less than two of these four MetS components are present [7], resembling absence of MetS (when the waist circumference component is omitted). However, Lavie et al. argue for a strict definition, where individuals with obesity classified as MHO should be fully healthy from a metabolic point of view, meaning that none of the four MetS components should be present [8,9]. Further, it is a common belief that individuals with MHO should have a similar risk of body mass index (BMI) related health problems, in particular cardiovascular events, as MH individuals with normal weight [7]. However, most studies show that MHO individuals have a higher risk for CVD compared to MH individuals with normal weight [7]. Following this, a new definition has been proposed [10], developed empirically through an extensive examination of various cardiometabolic risk factors and their association to CVD mortality and total mortality [10].

In this study, these three specific definitions were selected for comparison because they represent distinct and recognized approaches to defining MHO. The definition resembling MetS is the most widely used definition [7], while the strict version of the MetS based definition offers a more stringent perspective on defining MHO. The novel, empirical definition is based on an a posteriori data-driven approach, aiming to define MHO based on its association with clinical outcomes. This definition has shown evidence against a key criticism of the MHO concept, that MHO individuals are not truly healthy [11], as it identified MHO individuals not at increased risk of CVD and total mortality compared with MH normal weight individuals [10]. To our knowledge, no other studies have compared a definition resembling MetS, a strict definition, and the empiric definition in a general population with separate analyses for women and men. Stratifying results for women and men is thus a novel approach in this context, and important, as sex differences largely influence prevalence of MH and MU [3].

Using data from the population-based Tromsø Study, we examined and compared the prevalence of MH women and men according to three different definitions. We also analyzed the overlap between these definitions and examined the contribution of their individual components. Further, we described the metabolic and anthropometric characteristics of MHO individuals classified by the three definitions.

## Materials and methods

### Study population and data collection

The Tromsø Study is an ongoing population-based study with seven surveys to date (Tromsø1-Tromsø7, 1974–2016) conducted in Tromsø municipality, Norway [12,13], where total birth cohorts and random samples have been invited (65–79% attendance). Data collection includes questionnaires and interviews, biological sampling, and clinical examinations. All inhabitants in Tromsø aged 40 years and older were invited to Tromsø7 (2015–2016) (N = 32 591) and a total of 21 083 (65%) attended [12].

### Study sample

**Fig 1:**
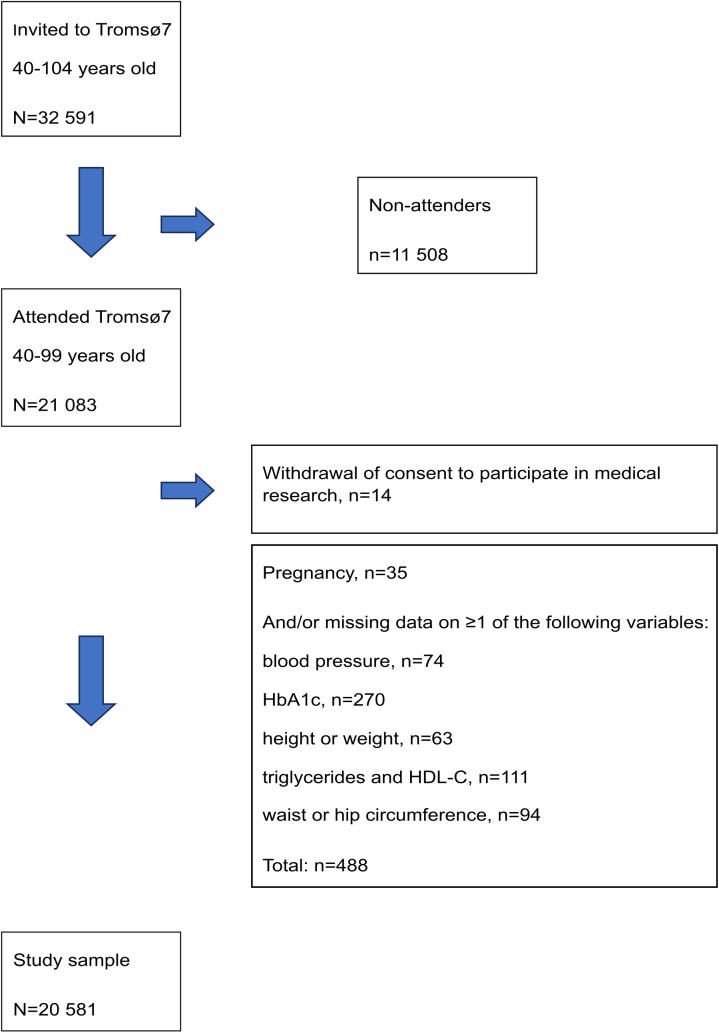
Flow chart of the study population. The Tromsø Study 2015-2016.

From the total sample of 11 074 women and 10 009 men 40–99 years old attending Tromsø7, we excluded participants who withdrew their consent to participate in medical research (n = 14). In addition, we excluded in total 488 participants based on current pregnancy (n = 35) and/or missing measurements of blood pressure (n = 74), glycated hemoglobin (HbA1c) (n = 270), height or weight (n = 63), serum triglycerides or HDL-C (n = 111), and waist- or hip circumference (n = 94). This resulted in a sample of 20 581 participants included in the analyses (Fig 1).

### Clinical examinations

Weight and height were measured with light clothing and no shoes with a Jenix DS-102 scale (DongSahn Jenix, Seoul, Korea). We calculated BMI by dividing weight in kilograms (kg) with the square of height in meters (m). Waist and hip circumference were measured with a Seca measurement tape at the level of the umbilicus and the greater trochanters, respectively. We calculated waist-to-hip ratio by dividing waist circumference by hip circumference in centimeters (cm). Systolic and diastolic blood pressure were measured on the right arm three times at 1-minute intervals after 2 minutes seated rest with a Dinamap ProCare 300 (GE Healthcare, Norway), and the mean of reading 2 and 3 was used in the analyses.

### Self-reported data from questionnaires

The following questions covered medication use: “Do you use, or have you used any of the following medications”: “cholesterol lowering drugs”, “blood pressure lowering drugs”, “insulin”, and “tablets for diabetes” (Never/Currently/Previously, not now). Diabetes status was assessed by: “Do you have, or have you had diabetes?” (No/Yes, now/Yes, previously), and pregnancy status by: “Are you pregnant now?” (No/Yes/Uncertain). For the classification of MH and MU, individuals with missing data on self-reported diabetes (n = 582) were classified as not having diabetes. Furthermore, individuals with missing data on use of blood pressure lowering drugs (n = 281), diabetes tablets (n = 487), cholesterol-lowering drugs (n = 393), and insulin (n = 393), were classified as nonusers.

### Blood sampling

Non-fasting venous blood samples were collected with standard methods by trained technicians and analyzed for HbA1c and serum concentrations of HDL-C and triglycerides at the Department of Laboratory Medicine at the University Hospital of North Norway (laboratory ISO certification NS-EN ISO 15189:2012).

### Definitions of metabolic health

We used three definitions of metabolic health status to categorize participants as MH or MU, called definition A (MetS), B (strict) or C (empiric) (Table 1). The criteria in both definition A and B are based on the harmonized ATP III definition of the metabolic syndrome (2009) [1], with some adjustments. Firstly, in accordance with previous practice [7] and the proposed definition by Lavie et al. [8,9], waist circumference is excluded. Secondly, the blood samples in Tromsø7 were collected non-fasting, while the ATP III definition specifies a cut-off for fasting glucose. To substitute this, we used HbA1c as a glycemic marker, with pre-diabetic levels as cut-off [14]. We also altered the cut-off for triglyceride levels from the ATP III definition, in accordance with that considered abnormal non-fasting triglyceride levels [15]. Finally, the data on medical treatment for triglycerides and HDL-C in Tromsø7 pertained to cholesterol-lowering drugs, which may not be directly comparable to the drug treatments specified in the harmonized ATP III criteria [1].

**Table 1:** Criteria for metabolic syndrome and three definitions of metabolic health status. The Tromsø Study 2015-2016.

Harmonized ATP III (2009) criteria for clinical diagnosis of the metabolic syndrome [1]	Definition A (MetS)	Definition B (strict)	Definition C (empiric)
Metabolic syndrome if fulfilling ≥3 of the following five components:	MH if fulfilling ≤1 of the following four components:	MH if fulfilling 0 of the following four components:	MH if fulfilling 0 of the following three components:
Elevated waist circumference (population- and country-specific definitions)			Waist-to-hip ratio ≥0.95 (women), ≥1.03 (men)
Triglycerides ≥1.7 mmol/l (fasting), or on treatment	Triglycerides ≥2.00 mmol/l (non-fasting), and/or medication use	Triglycerides ≥2.00 mmol/l (non-fasting), and/or medication use	
HDL-C <1.0 mmol/l (men), <1.30 mmol/l (women), or on treatment	HDL-C <1.00 mmol/l (men), <1.30 mmol/l (women), and/or medication use	HDL-C <1.00 mmol/l (men), <1.30 mmol/l (women), and/or medication use	
Blood pressure ≥130/85 mmHg, or on treatment	Blood pressure ≥130/85 mmHg, and/or medication use	Blood pressure ≥130/85 mmHg, and/or medication use	Systolic blood pressure ≥130 mmHg, and/or medication use
Fasting glucose ≥100 mg/dL, or on treatment	HbA1c ≥6.0%, and/or self-reported diabetes, and/or medication use	HbA1c ≥6.0%, and/or self-reported diabetes, and/or medication use	Self-reported diabetes, and/or HbA1c ≥6.5%

An overview of the harmonized ATP III criteria for metabolic syndrome as well as the three definitions used to classify individuals’ metabolic health status in this study. ATP III; Adult Treatment Panel III, MH; Metabolically healthy.

Thus, the following four criteria and cut offs were used to classify MU in definition A and B: 1) systolic blood pressure ≥130 mmHg and/or diastolic blood pressure ≥85 mmHg, and/or self-reported current use of blood pressure lowering drugs, 2) HDL-C <1.30 mmol/l in women and <1.0 mmol/l in men, and/or self-reported current use of cholesterol-lowering drugs, 3) non-fasting triglyceride levels ≥2.0 mmol/l, and/or self-reported current use of cholesterol-lowering drugs, 4) HbA1c ≥6.0%, and/or self-reported diabetes, and/or self-reported current use of diabetes tablets or insulin.

What separates definition A and B is the number of components fulfilled to classify individuals as MH or MU. In definition A, ≥2 of the four MetS components must be fulfilled to be MU (the common version), while in definition B, only ≥1 of the four MetS components must be fulfilled (the strict version suggested by Lavie et al.) [8,9].

Definition C was based on the empirically derived definition by Zembic et al. [10] where participants with ≥1 of the following three components were categorized as MU: 1) systolic blood pressure ≥130 mmHg, and/or self-reported use of blood pressure lowering drugs, 2) self-reported diabetes, and/or HbA1c ≥6.5%, 3) waist-to-hip ratio ≥0.95 (women), ≥1.03 (men). We added HbA1c levels ≥6.5% to the original definition to also include individuals with undiagnosed diabetes.

Those not classified as MU, were considered MH.

### Statistical analyses

Statistical analyses were performed using Stata (StataCorp. 2023. Stata Statistical Software: Release 18. College Station, TX: StataCorp LLC). Participants were categorized according to BMI as having normal weight (BMI <25 kg/m^2^), overweight (BMI ≥25–29.9 kg/m^2^) or obesity (BMI ≥30 kg/m^2^). Thus, participants with underweight (BMI <18.5 kg/m^2^) (<0.6% [n = 114] of the analytical sample) were included in the normal weight BMI category. To ensure the robustness of our findings, sensitivity analyses were conducted excluding participants with BMI <18.5 kg/m^2^.

We used descriptive analyses to assess the prevalence of MH women and men in BMI categories according to definition A (MetS), B (strict), and C (empiric). Moreover, we assessed which component was fulfilled by those categorized as MH according to definition A (MetS), but as MU with definition B (strict), i.e., individuals fulfilling only one MetS component.

To assess potential differences in metabolic risk factors and anthropometric characteristics between the MH and MU participants with obesity according to the three definitions, we presented age-adjusted mean values with 95% confidence intervals (CIs) for normally distributed variables (HbA1c, diastolic and systolic blood pressure, HDL-C, waist circumference, BMI, waist-to-hip ratio). Assessment of normal distribution was performed by visual inspection of histograms for participants in the main study sample (n = 20 581). Triglyceride levels were non-normally distributed; thus, they were log10-transformed to calculate the geometric mean with 95% CIs, and the resulting age-adjusted means with 95% CIs were then back transformed (exponentiating by 10). The mean levels of the metabolic and anthropometric characteristics were age-adjusted using linear regression and presented using the mean age of women and men with obesity in the sample (57.0 years).

The Venn diagrams providing a visual representation of the overlap and difference between the three definitions in classifying MH individuals, as well as the fulfillment of components in MU women and men for each definition, were made in R (v4.3.1, R Core Team, 2023) [16] using the nVennR package [17].

### Ethics

This study is approved by the Regional Committee for Medical Research Ethics Northern Norway, REK North. The participants provided their written informed consent to participate in the study. Data from Tromsø7 used in this study was received and accessed 27/6/2022. The authors did not have access to information that could directly identify individual participants during or after data collection of Tromsø7. However, since the dataset includes a large range of variables per individual, there is a theoretical possibility for reverse identification of participants.

## Results

Characteristics of the study sample are shown in Table 2. The proportion of women was 52%. Based on BMI, most women had either normal weight (40%) or overweight (37%). Twenty-two percent had obesity. Among men, 51% had overweight, with about similar proportions having normal weight (24%) and obesity (25%). About half of all participants fulfilled the blood pressure component included in the three definitions.

**Table 2:** Characteristics of participants: The Tromsø Study 2015–2016.

	Women	Men	Total
	52%	48%	
	n = 10 773	n = 9 808	N = 20 581
**Age groups (%, n)**			
40–49	31 (3 291)	31 (2 995)	31 (6 286)
50–59	29 (3 176)	28 (2 735)	29 (5 911)
60–69	24 (2 599)	25 (2 461)	25 (5 060)
70–79	12 (1 315)	13 (1 286)	13 (2 601)
80+	4 (392)	3 (331)	4 (723)
**Anthropometric characteristics:**			
**BMI categories, kg/m** ^**2**^ **(%,n)**			
Normal weight, <25	40 (4 351)	24 (2 365)	33 (6 716)
Overweight, 25–29.9	37 (4 006)	51 (4 973)	44 (8 979)
Obesity, ≥30	22 (2 416)	25 (2 470)	24 (4 886)
**BMI, kg/m**^**2**^ **(mean, SD)**	26.9 (4.9)	27.8 (4.0)	27.3 (4.5)
**Waist circumference, cm (mean, SD)**	90.7 (12.9)	100.2 (11.2)	95.2 (13.0)
**Hip circumference, cm (mean, SD)**	103.8 (9.8)	104.3 (6.9)	104.0 (8.5)
**Waist-to-hip ratio (mean, SD)**	0.87 (0.08)	0.96 (0.07)	0.91 (0.09)
**Metabolic characteristics:**			
**Systolic blood pressure, mmHg (mean, SD)**	126.9 (20.8)	132.6 (18.1)	129.6 (19.8)
**Diastolic blood pressure, mmHg (mean, SD)**	72.8 (9.7)	78.3 (9.7)	75.4 (10.1)
**Triglycerides, mmol/l (mean, SD)**	1.35 (0.75)	1.72 (1.08)	1.53 (0.94)
**HDL-C, mmol/l (mean, SD)**	1.73 (0.49)	1.39 (0.40)	1.57 (0.48)
**HbA1c, % (mean, SD)**	5.7 (0.6)	5.7 (0.7)	5.7 (0.6)
**Medication use (%, n):**			
**Use of blood pressure lowering drugs**			
Currently	21 (2 191)	24 (2 334)	22 (4 525)
Previously	3 (296)	3 (254)	3 (550)
Never	77 (8 140)	73 (7 085)	75 (15 225)
**Use of cholesterol-lowering drugs**			
Currently	13 (1 348)	17 (1 669)	15 (3 017)
Previously	3 (305)	3 (308)	3 (613)
Never	84 (8 888)	80 (7 670)	82 (16 558)
**Use of tablets for diabetes**			
Currently	3 (300)	4 (398)	3 (698)
Previously	1 (63)	0 (47)	1 (110)
Never	97 (10 122)	95 (9 164)	96 (19 286)
**Use of insulin**			
Currently	1 (125)	2 (178)	2 (303)
Previously	0 (36)	0 (22)	0 (58)
Never	98 (10 275)	98 (9 359)	98 (19 634)
**Self-reported diabetes (%, n)**			
No	95 (9 918)	94 (8 996)	95 (18 914)
Yes	4 (442)	6 (532)	5 (974)
Previously	1 (65)	0 (46)	1 (111)
**Fulfilling the component for MU according to (%, n):**			
**Definition A and B:**			
Blood pressure^1^	46 (5 006)	61 (5 994)	53 (11 000)
Triglycerides^2^	26 (2 752)	41 (4 057)	33 (6 809)
HDL-C^3^	26 (2 806)	26 (2 519)	26 (5 325)
HbA1c^4^	16 (1 696)	18 (1 786)	17 (3 482)
**Definition C:**			
Blood pressure^5^	46 (4 910)	59 (5 825)	52 (10 735)
Diabetes ^6^	5 (576)	8 (741)	6 (1 317)
Waist-to-hip ratio^7^	15 (1 575)	15 (1 487)	15 (3 062)

Data is presented as % (number) and mean (SD) where appropriate. ^**1**:^ Systolic blood pressure ≥130 mmHg, and/or diastolic blood pressure ≥85 mmHg, and/or self-reported current use of blood pressure lowering drugs ^2:^ Non-fasting triglyceride levels ≥2.0 mmol/l, and/or self-reported current use of cholesterol-lowering drugs ^3:^ HDL-C <1.30 mmol/l (women) and <1.0 mmol/ (men), and/or self-reported current use of cholesterol-lowering drugs ^4:^ HbA1c ≥6.0%, and/or self-reported diabetes, and/or self-reported current use of diabetes tablets or insulin ^5:^ Systolic blood pressure ≥130 mmHg, and/or self-reported current use of blood pressure lowering drugs ^6:^ HbA1c ≥6.5%, and/or self-reported diabetes ^7:^ Waist-to-hip ratio ≥0.95 (women), ≥1.03 (men). N = 20 581. Data were not complete for self-reported diabetes (n = 582) and for the following medications: Blood pressure lowering drugs (n = 281), diabetes tablets (n = 487), cholesterol-lowering drugs (n = 393), and use of insulin (n = 393). Missing data are not included in the table.

**Fig 2:**
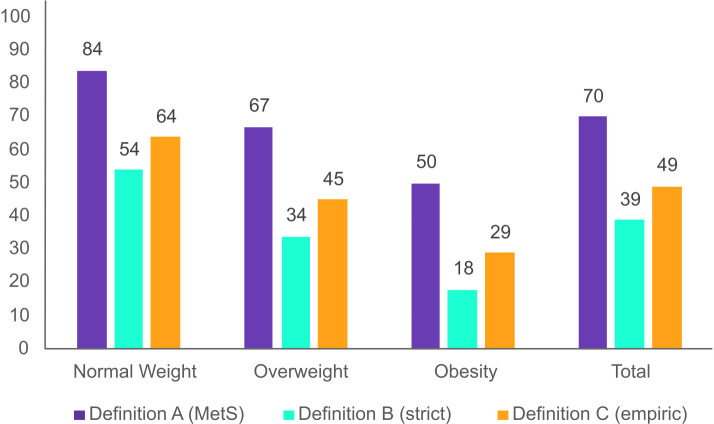
Proportion of metabolically healthy women according to three definitions. The Tromsø Study 2015-2016. Definition A (MetS); Metabolically healthy if fulfilling ≤1 out of 4 metabolic syndrome components. Definition B (strict); Metabolically healthy if fulfilling 0 out of 4 metabolic syndrome components. Definition C (empiric); Metabolically healthy if fulfilling 0 out of 3 components including waist-to-hip ratio, systolic blood pressure and diabetes.

**Fig 3:**
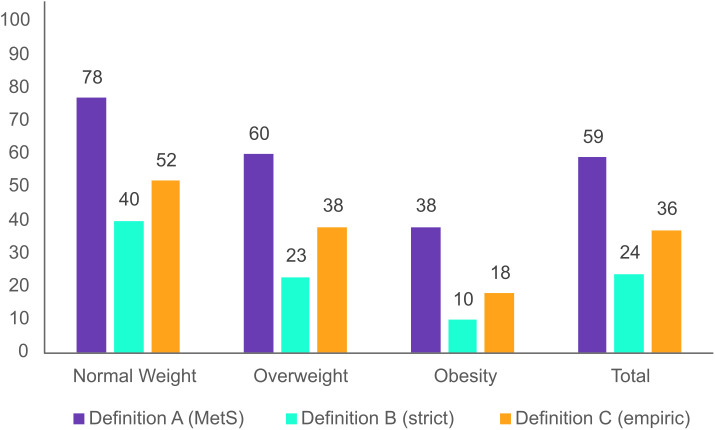
Proportion of metabolically healthy men according to three definitions. The Tromsø Study 2015-2016. Definition A (MetS); Metabolically healthy if fulfilling ≤1 out of 4 metabolic syndrome components. Definition B (strict); Metabolically healthy if fulfilling 0 out of 4 metabolic syndrome components. Definition C (empiric); Metabolically healthy if fulfilling 0 out of 3 components including waist-to-hip ratio, systolic blood pressure and diabetes.

Overall, the proportion of MH, according to each definition, was higher in women than in men (p <0.001). Further, the proportion of MH individuals differed by BMI category (p <0.001) in both women and men, with higher proportion of MH observed in the lower BMI categories (Figs 2 and 3) (results for the conducted chi-square tests not shown). Definition A yielded the highest proportion of MH across all BMI categories, whereas Definition B resulted in the lowest. In contrast, Definition C produced MH prevalences that fell between those of Definitions A and B across all BMI categories. A total of 50% women and 38% men had MHO based on definition A (MetS). With definition B (strict), the prevalences of MHO dropped to 18% for women and 10% for men. Definition C (empiric) showed intermediate prevalences of MHO, with 29% for women and 18% for men.

**Fig 4:**
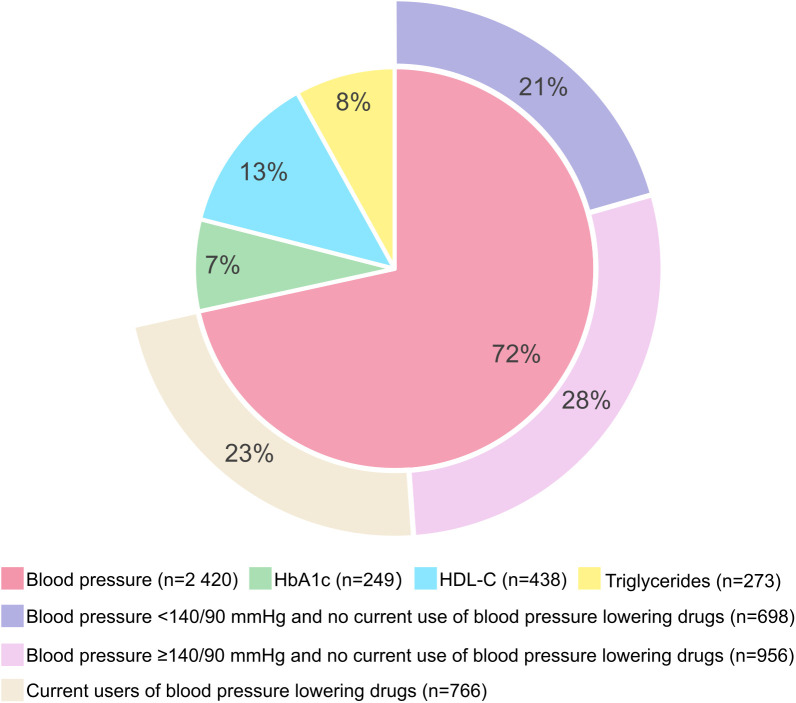
Distribution of MetS components in women fulfilling only one MetS component. The Tromsø Study 2015-2016. The inner circle represents the distribution of the four metabolic syndrome components in women fulfilling only one metabolic syndrome component: Blood pressure; Systolic blood pressure ≥130 mmHg and/or diastolic blood pressure ≥85 mmHg, and/or self-reported current use of blood pressure lowering drugs. HbA1c; HbA1c ≥6.0%, and/or self-reported diabetes, and/or self-reported current use of diabetes tablets or insulin. HDL-C; HDL-C <1.30 mmol/l, and/or self-reported current use of cholesterol-lowering drugs. Triglycerides; Non-fasting triglyceride levels ≥2.0 mmol/l, and/or self-reported current use of cholesterol-lowering drugs. The outer circle represents the proportion of women with blood pressure levels above and below the cut-off for hypertension (blood pressure ≥140/90 mmHg) and use of blood pressure lowering drugs, in women fulfilling the blood pressure component only.

**Fig 5:**
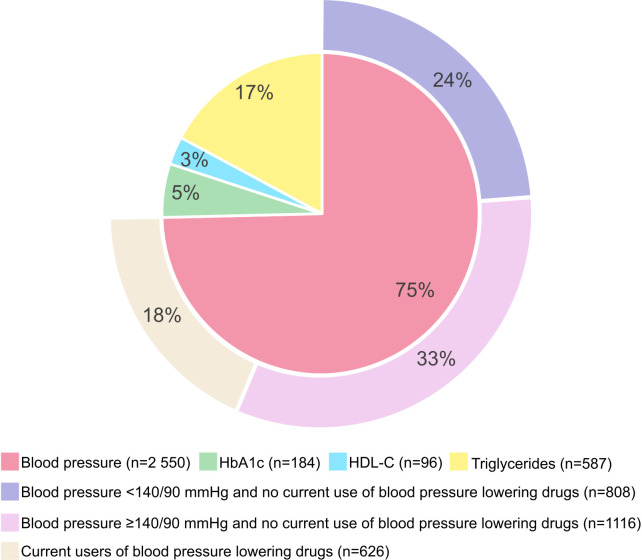
Distribution of MetS components in men fulfilling only one MetS component. The Tromsø Study 2015-2016. The inner circle represents the distribution of the four metabolic syndrome components in men fulfilling only one metabolic syndrome component: Blood pressure; Systolic blood pressure ≥130 mmHg and/or diastolic blood pressure ≥85 mmHg, and/or self-reported current use of blood pressure lowering drugs. HbA1c; HbA1c ≥6.0%, and/or self-reported diabetes, and/or self-reported current use of diabetes tablets or insulin. HDL-C; HDL-C <1.0 mmol, and/or self-reported current use of cholesterol-lowering drugs. Triglycerides; Non-fasting triglyceride levels ≥2.0 mmol/l, and/or self-reported current use of cholesterol-lowering drugs. The outer circle represents the proportion of men with blood pressure levels above and below the cut-off for hypertension (blood pressure ≥140/90 mmHg) and use of blood pressure lowering drugs, in men fulfilling the blood pressure component only.

Relatively many of the participants who were classified as MH according to Definition A and MU under Definition B, i.e., fulfilling only one MetS component, fulfilled the blood pressure component, primarily due to elevated levels without use of blood pressure lowering drugs. Among those who fulfilled only one MetS component, 28% of the women and 33% of the men had blood pressure levels above the hypertensive threshold (without use of blood pressure lowering drugs). However, more than 20% of women and men who fulfilled only one MetS component had levels below the clinical criteria for hypertension (blood pressure ≥140/90 mmHg), but fulfilled the blood pressure component due to levels above 130/85 mmHg without use of blood pressure lowering drugs (Figs 4 and 5).

In sub-analyses, we assessed this distribution in different BMI categories and observed similar patterns (results not shown).

**Fig 6:**
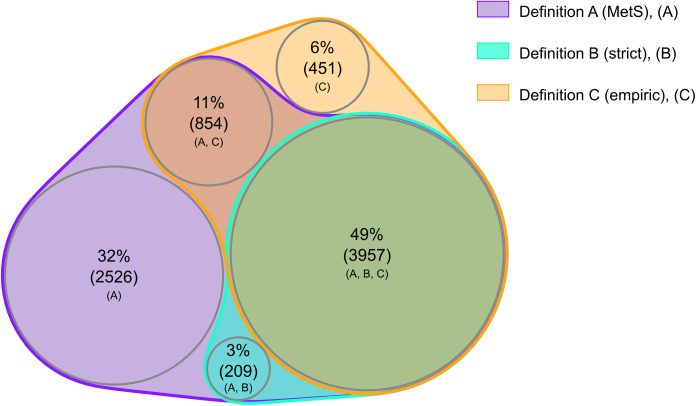
Proportion and overlap of MH women according to three definitions. The Tromsø Study 2015-2016. Definition A (MetS); Metabolically healthy if fulfilling ≤1 out of 4 metabolic syndrome components. Definition B (strict); Metabolically healthy if fulfilling 0 out of 4 metabolic syndrome components. Definition C (empiric); Metabolically healthy if fulfilling 0 out of 3 components including waist-to-hip ratio, systolic blood pressure and diabetes.

**Fig 7:**
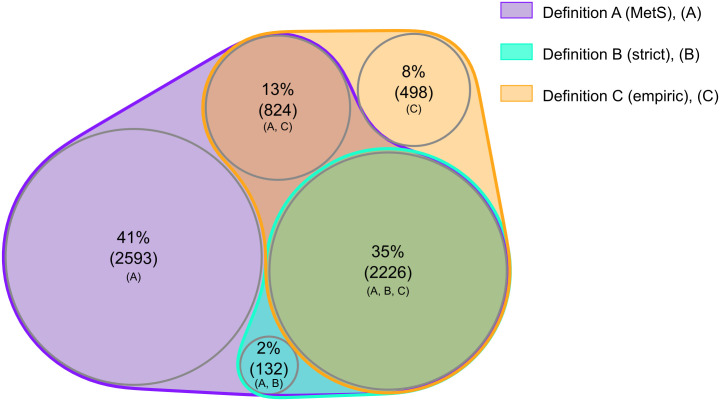
Proportion and overlap of MH men according to three definitions. The Tromsø Study 2015-2016. Definition A (MetS); Metabolically healthy if fulfilling ≤1 out of 4 metabolic syndrome components. Definition B (strict); Metabolically healthy if fulfilling 0 out of 4 metabolic syndrome components. Definition C (empiric); Metabolically healthy if fulfilling 0 out of 3 components including waist-to-hip ratio, systolic blood pressure and diabetes.

A total of 49% of MH women and 35% of MH men were classified as MH by all three definitions (Figs 6 and 7). Since definition A classifies both those with none and one MetS component as MH, it substantially overlaps with the MH classifications using definition B and C. A total of 32% of MH women and 41% of MH men were classified as MH according to definition A alone. Most of the individuals classified as MH according to definition B (strict) were also MH according to definition C (empiric). However, definition C included additional 1305 women and 1322 men compared to definition B. Only 6% of MH women and 8% of MH men were classified as MH according to definition C alone.

Overall, the most frequently fulfilled component in MU women and men was blood pressure. When using definitions A, B and C, this component was respectively met by 80%, 76%, and 89% of MU women, and 86%, 81%, and 93% of MU men. Additionally, more than a third of MU participants according to definition B and two thirds according to definition C were classified as MU due to fulfilling the blood pressure component alone (S1 Fig).

Anthropometric and metabolic characteristics in MH and MU women and men with obesity according to the three definitions are shown in S2 Table. MHO participants had favorable levels of all the metabolic and anthropometric characteristics compared to MUO participants classified with the same definition. Further, MHO individuals according to definition B (strict) generally had more favorable levels of all the characteristics compared to MHO individuals according to definition A (MetS). When using definition C (empiric), MHO participants had lower systolic and diastolic blood pressure and anthropometric measures, while unfavorable levels of the other metabolic characteristics compared to definition A (MetS). Similar results were observed when comparing MHO in definition C (empiric) with definition B (strict), although the levels of systolic and diastolic blood pressure were more similar.

Sensitivity analyses excluding participants with a BMI <18.5 kg/m², yielded results similar to those obtained when these participants were included (results not shown).

## Discussion

In this population-based study, we assessed the prevalence of MH according to three definitions: definition A (MetS); ≤1 out of 4 MetS components; definition B (strict); 0 out of 4 MetS components; and definition C (empiric): 0 out of 3 components including systolic blood pressure, waist-to-hip ratio and diabetes. The prevalence of MH was higher in women compared to men and lower in higher BMI categories in both women and men. The proportion of MH individuals varied considerably depending on the definition used. In individuals with obesity, the prevalence of MH was 50% in women and 38% in men according to definition A (MetS). However, using definition B (strict), the prevalence of MH was considerably lower, at 18% for women and 10% for men. This difference in prevalences of MH was primarily attributed to many participants only fulfilling the blood pressure component. Definition C (empiric) resulted in prevalences of MH between those observed by the other definitions, with 29% and 18% for women and men, respectively.

A review summarizing results from 25 studies showed that about half of individuals with obesity were MH according to a definition resembling MetS [18]. Further, according to studies using a strict definition requiring that none of the MetS components were present, the overall prevalence of MHO was about 13%. Although these combined prevalences differ from our results, the review presents a large range of prevalences from different studies. Our results were comparable to several of the prevalences from the single studies included in the review. The authors proposed that the prevalence of MHO might be influenced by variations across studies in factors such as sex, age, BMI range, ethnicity, and the exclusion of individuals with existing cardiometabolic conditions like CVD and diabetes [18]. In addition, it should be noted that using different parameters and cut-offs also leads to different prevalences and hampers the comparability between studies. Notably, when a unified criteria resembling MetS was used to compare prevalences in different European populations, the prevalence of MHO varied from 24–65% in women and from 43–78% in men [19]. This could be due to study or population differences but might also indicate that the prevalence of MHO differs in different populations. The prevalence of MHO when using definition C (empiric) in our study was 29% and 18% in women and men, respectively. Other studies using the same definition to classify MHO found prevalences between 17–41% in populations in Europe [10,20,21], Asia [22,23] and the Middle East [24].

The mechanisms that enable some individuals to remain metabolically healthy despite living with obesity are not yet fully understood and are beyond the scope of this article. However, it is widely recognized that distribution and function of adipose tissue play a central role [2,6,7,11,25]. The higher prevalence of MH women compared to men found in this study, which is in accordance with findings from previous studies [3,19,26], could thus possibly be explained by sex differences in body fat distribution. In addition, other behavioral or sociodemographic factors such as physical activity, diet, smoking, alcohol consumption and socioeconomic status are also known to be associated with cardiometabolic health [27–31] and could potentially explain the differences in prevalence of MH between women and men. However, this warrants further investigation.

Elevated blood pressure was the most common component contributing to MU classification across all three definitions. It may be worth discussing whether this component alone should suffice for MU classification. Many fulfilling only the blood pressure component used in definitions A (MetS) and B (strict) had levels below the hypertensive threshold of 140/90 mmHg set by the 2024 European Society of Cardiology guidelines [32], without use of medication. However, the association between blood pressure and adverse CVD outcomes is continuous and log-linear, with elevated risk for blood pressure levels also below the cut-off of ≥140/90 mmHg [32]. Due to the continuous relationship, defining an optimal cut-off to differentiate between truly metabolically healthy and unhealthy individuals is challenging. Other cut-offs for blood pressure, such as 140/90 mmHg, 135/85 mmHg, and 120/80 mmHg have also been used to define MH individuals in previous studies [5]. However, the cut-off of 130/85 mmHg, which is included in the harmonized ATP III definition of the metabolic syndrome (2009) [1], is the most common [5]. Thus, this was used in definitions A (MetS) and B (strict) in this study. In addition, the empirically derived definition by Zembic et al. (definition C in this study) included systolic blood pressure, with a cut-off of 130 mmHg. This cut-off was chosen by Zembic et al. as it closely aligned with the statistically optimal values identified using the Youden Index for predicting both CVD mortality (125 mmHg) and total mortality (124 mmHg), while also adhering to established clinical guidelines for pre-hypertension [10].

There is an ongoing debate as to whether the MHO concept represents a truly healthy phenotype protected from cardiometabolic events and chronic diseases [33,34]. According to a recent review, most MHO individuals have higher CVD risk than MH normal weight individuals when using different definitions based on MetS or insulin resistance [7]. Since definitions resembling MetS allow presence of up to two components (when using five MetS components including waist circumference), and still being considered MH, there is a possibility that MH individuals with obesity and normal weight could differ in numbers of components. This means that MHO individuals might have both diabetes and elevated blood pressure while MH normal weight individuals might have none of the components. This could explain the observed higher CVD risk in MHO individuals compared to MH normal weight individuals. Both meta-analyses and cohort studies using stricter definitions, where MH classification requires absence of any metabolic risk factor, have provided conflicting results [35–39,40]. The number of studies assessing health risk for MHO individuals is still sparse, and the selected parameters and the strictness for the cut-offs varies considerably between the studies. Therefore, it is challenging to draw a firm conclusion regarding health risk of MHO defined by strict definitions.

Contrary to previous definitions of MHO, which are based on a priori assumptions of what constitutes MH, the definition by Zembic et al. was derived empirically by systematic assessment of a broad range of risk factors with CVD and total mortality [7,10]. The empiric definition was superior to distinguish at-risk and not-at-risk individuals, compared to a definition resembling MetS and a definition based on insulin resistance. Moreover, the empiric definition showed that MHO individuals were not at increased risk of CVD and total mortality, compared with MH normal weight individuals [10]. To date, several studies have found the empiric definition to identify MHO individuals without increased risk for total mortality [10,20,22], stroke and CVD events [21,22], compared to MH individuals with normal weight. However, a study from China found that MHO individuals had increased risk for coronary heart disease compared to MH normal weight individuals [22]. More studies with longer follow-up are needed to confirm that the empiric definition identifies MHO individuals without increased health risk.

We present mean levels of metabolic and anthropometric risk factors in MHO individuals classified by definition A (MetS), B (strict) and C (empiric). Individuals classified as MHO by definition C (empiric) had higher mean HbA1c, triglycerides and lower mean HDL-C, but lower mean blood pressure, waist circumference, waist-to-hip ratio and BMI compared to definition A (MetS). These results align with the findings from a study from China comparing MHO individuals defined by the empiric definition and a definition resembling MetS [22]. Unlike our results, they found that empirically defined MHO individuals had higher anthropometric measurements than those defined by the MetS resembling criteria. They further showed that empirically defined MHO individuals had a similar CVD risk to metabolically healthy normal weight individuals, whereas MetS defined MHO individuals had a higher CVD risk. The authors suggested that the differing CVD risks could be due to lower blood pressure in empirically defined MHO individuals. In our study, MHO individuals classified by definition B (strict) had a more favorable risk factor profile compared to those classified by definition A (MetS). This could suggest that a stricter definition might better identify MHO individuals without increased long-term health risks than the more commonly used MetS definition. However, other risk factors not included in our, nor the study from China [22], might also influence the association between metabolic health and CVD. This could be factors such as body composition, low-density lipoprotein cholesterol and inflammatory markers, which were beyond the scope of this article.

The considerable difference in prevalence of MH when using the three definitions highlights the need for a consensus definition. A major issue is the lack of consensus concerning what MHO should represent. The question is whether it should represent individuals without cardiometabolic risk factors now or over time, who MHO individuals should be compared with, as well as to how strict the criteria used should be. For example, it has recently been proposed that MHO could be defined by absence of hospitalization for several decades in mid-life [41]. A broad understanding of what MHO represents will lead to different criteria resulting in different prevalences. If MHO individuals and MH normal weight individuals have similar risks for CVD, we lack sufficient evidence for such definition. The empirical definition by Zembic et al. [10] appears promising compared to traditional definitions, although more evidence is needed. The empiric definition includes waist-to-hip ratio, which reflects adipose tissue distribution. Differences in adipose tissue distribution is proposed to be responsible for the difference in metabolic health between MH and MU individuals with obesity [18].

The lower prevalence of MH in higher BMI categories supports measures promoting non-obesogenic societies. However, at an individual level, more knowledge regarding the long-term effects of MHO could lead to more personalized approaches in treatment and prevention strategies. Although most studies show that MHO individuals have higher CVD risk compared to MH normal weight individuals, it is well documented that MHO individuals have lower risk for CVD compared to their MU counterparts [7]. This highlights the importance of identifying individuals in most need of preventive measures.

Strengths of this study include the use of a large population-based sample with high attendance (65%), making our results representative for similar populations with similar living conditions and lifestyle. Moreover, data collection was performed using standard procedures and validated questionnaires. All physical examinations were performed by trained personnel, enhancing the likelihood that we obtain accurate data and valid results.

However, there are also some limitations. Firstly, our results may not be representative of younger age groups (i.e., < 40 years) or ethnicities not prevalent in the Tromsø municipality; the large majority is white individuals. Secondly, although we have high quality data concerning metabolic health (for example measured, not self-reported, weight, height and blood pressure), we acknowledge that it would have strengthened our study if we had similar data for direct comparison to previous studies (i.e., fasting blood samples). Third, the use of self-reported medication data introduces a potential source of bias, as self-reporting may be subject to recall errors or misclassification. Our approach to handling missing data, such as treating missing values as non-users of medication, could also bias the classification of participants and influence the results. However, sensitivity analyses excluding individuals with missing data on medication use and self-reported diabetes, did not lead to meaningful changes in the results, nor the conclusions. Lastly, other common definitions and parameters used for MHO classification, such as those based on insulin sensitivity (e.g., homeostatic model assessment-insulin resistance (HOMA-IR), euglycemic-hyperinsulinemic clamp, or oral glucose tolerance test), could not be included in the present study as we did not have such data.

## Conclusion

The prevalence of MH differs substantially based on the definition used. A consensus concerning what MHO represents, and a better understanding of the long-term health risks and the related mechanisms are needed to establish a universal definition and targeted preventive efforts. Further, we recommend that future studies validate the empiric definition in large cohorts to establish to what extent this definition identifies truly metabolically healthy individuals.

## Supporting information

S1 Table Proportion of metabolically healthy and unhealthy participants according to three different definitions of metabolic health in categories of body mass index. The Tromsø Study 2015–2016. Definition A (MetS); Metabolically healthy by fulfilling ≤1 out of 4 metabolic syndrome components, and metabolically unhealthy by fulfilling ≥2 out of 4 metabolic syndrome components. Definition B (strict); Metabolically healthy by fulfilling 0 out of 4 metabolic syndrome components, and metabolically unhealthy by fulfilling ≥1 out of 4 metabolic syndrome components. Definition C (empiric); Metabolically healthy by fulfilling 0 out of 3 components including waist-to-hip ratio, systolic blood pressure and diabetes, and metabolically unhealthy by fulfilling ≥1 of the 3 components. Data is presented as X% (number). N = 20 581. Abbreviations: MH; metabolically healthy, MU; metabolically unhealthy, BMI; Body mass index.
(DOCX)

S2 Table Mean age-adjusted levels of anthropometric and metabolic characteristics in MHO and MUO women and men by three definitions of metabolic health. The Tromsø Study 2015–2016. Definition A (MetS); Metabolically healthy by fulfilling ≤1 out of 4 metabolic syndrome components, and metabolically unhealthy by fulfilling ≥2 out of 4 metabolic syndrome components. Definition B (strict); Metabolically healthy by fulfilling 0 out of 4 metabolic syndrome components, and metabolically unhealthy by fulfilling ≥1 out of 4 metabolic syndrome components. Definition C (empiric); Metabolically healthy by fulfilling 0 out of 3 components including waist-to-hip ratio, systolic blood pressure and diabetes, and metabolically unhealthy by fulfilling ≥1 of the 3 components. Abbreviations: MHO; Metabolically healthy obesity, MUO; Metabolically unhealthy obesity, BMI; Body mass index, HDL-C; High-density lipoprotein cholesterol. The values represent age-adjusted mean levels (95% confidence intervals).
(DOCX)

S1 Fig Venn diagrams representing fulfilled components in metabolically unhealthy women and men. The Tromsø study 2015–2016. Definition A (MetS); Metabolically unhealthy by fulfilling ≥2 out of 4 metabolic syndrome components. Definition B (strict); Metabolically unhealthy by fulfilling ≥1 out of 4 metabolic syndrome components. Definition C (empiric); Metabolically unhealthy by fulfilling ≥1 out of 3 components including waist-to-hip ratio, systolic blood pressure and diabetes. Blood pressure (Definition A and B); Systolic blood pressure ≥130 mmHg and/or diastolic blood pressure ≥85 mmHg, and/or self-reported current use of blood pressure lowering drugs. HbA1c; HbA1c ≥6.0%, and/or self-reported diabetes, and/or self-reported current use of diabetes tablets or insulin. HDL-C; HDL-C <1.30 mmol/l (women) and <1.0 mmol/ (men), and/or self-reported current use of cholesterol-lowering drugs. Triglycerides; Non-fasting triglyceride levels ≥2.0 mmol/l, and/or self-reported current use of cholesterol-lowering drugs. Blood pressure (Definition C); Systolic blood pressure ≥130 mmHg, and/or self-reported current use of blood pressure lowering drugs. Diabetes; HbA1c ≥6.5%, and/or self-reported diabetes. Waist-to-hip ratio; Waist-to-hip ratio ≥0.95 (women), ≥1.03 (men).
(TIF)
