# Supplementary material for: Assessing metabolic health in a general population: A comparative analysis of three definitions in the Tromsø Study 2015–2016
Source: PLoS One. 2025 Oct 6;20(10):e0333402. doi: 10.1371/journal.pone.0333402 (PMC12500164; doi:10.1371/journal.pone.0333402)
Supplement: S1 Table — The Tromsø Study 2015–2016. Definition A (MetS); Metabolically healthy by fulfilling ≤1 out of 4 metabolic syndrome components, and metabolically unhealthy by fulfilling ≥2 out of 4 metabolic syndrome components. Definition B (strict); Metabolically healthy by fulfilling 0 out of 4 metabolic syndrome components, and metabolically unhealthy by fulfilling ≥1 out of 4 metabolic syndrome components. Definition C (empiric); Metabolically healthy by fulfilling 0 out of 3 components including waist-to-hip ratio, systolic blood pressure and diabetes, and metabolically unhealthy by fulfilling ≥1 of the 3 components. Data is presented as X% (number). N = 20 581. Abbreviations: MH; metabolically healthy, MU; metabolically unhealthy, BMI; Body mass index. (DOCX) [file pone.0333402.s001.docx]

| **S1 Table: Proportion of metabolically healthy and unhealthy participants according to three different definitions of metabolic health in categories of body mass index. The Tromsø Study 2015-2016** | | | | | | | | |
| --- | --- | --- | --- | --- | --- | --- | --- | --- |
|  | **Normal weight (BMI <25 kg/m^2^)** | | **Overweight**  **(BMI 25-29.9 kg/m^2^)** | | **Obesity**  **(BMI ≥30 kg/m^2^)** | | **Total** | |
| **Women** | n=4 351 | | n=4 006 | | n=2 416 | | n=10 773 | |
|  | **MH** | **MU** | **MH** | **MU** | **MH** | **MU** | **MH** | **MU** |
| Definition A (MetS) | 84 (3 642) | 16 (709) | 67 (2 687) | 33 (1 319) | 50 (1 217) | 50 (1 199) | 70 (7 546) | 30 (3 227) |
| Definition B (strict) | 54 (2 345) | 46 (2 006) | 34 (1 379) | 66 (2 627) | 18 (442) | 82 (1 974) | 39 (4 166) | 61 (6 607) |
| Definition C (empiric) | 64 (2 770) | 36 (1 581) | 45 (1 800) | 55 (2 206) | 29 (692) | 71 (1 724) | 49 (5 262) | 51 (5 511) |
| **Men** | n=2 365 | | n=4 973 | | n=2 470 | | n=9 808 | |
| Definition A (MetS) | 78 (1 836) | 22 (529) | 60 (2 989) | 40 (1 984) | 38 (950) | 62 (1 520) | 59 (5 775) | 41 (4 033) |
| Definition B (strict) | 40 (946) | 60 (1 419) | 23 (1 161) | 77 (3 812) | 10 (251) | 90 (2 219) | 24 (2 358) | 76 (7 450) |
| Definition C (empiric) | 52 (1 219) | 48 (1 146) | 38 (1 886) | 62 (3 087) | 18 (443) | 82 (2 027) | 36 (3 548) | 64 (6 260) |
| Definition A (MetS); Metabolically healthy by fulfilling ≤1 out of 4 metabolic syndrome components, and metabolically unhealthy by fulfilling ≥2 out of 4 metabolic syndrome components. Definition B (strict); Metabolically healthy by fulfilling 0 out of 4 metabolic syndrome components, and metabolically unhealthy by fulfilling ≥1 out of 4 metabolic syndrome components. Definition C (empiric); Metabolically healthy by fulfilling 0 out of 3 components including waist-to-hip ratio, systolic blood pressure and diabetes, and metabolically unhealthy by fulfilling ≥1 of the 3 components. Data is presented as X% (number). N=20 581. Abbreviations: MH; metabolically healthy, MU; metabolically unhealthy, BMI; Body mass index | | | | | | | | |
