# Supplementary material for: Assessing metabolic health in a general population: A comparative analysis of three definitions in the Tromsø Study 2015–2016
Source: PLoS One. 2025 Oct 6;20(10):e0333402. doi: 10.1371/journal.pone.0333402 (PMC12500164; doi:10.1371/journal.pone.0333402)
Supplement: S2 Table — The Tromsø Study 2015–2016. Definition A (MetS); Metabolically healthy by fulfilling ≤1 out of 4 metabolic syndrome components, and metabolically unhealthy by fulfilling ≥2 out of 4 metabolic syndrome components. Definition B (strict); Metabolically healthy by fulfilling 0 out of 4 metabolic syndrome components, and metabolically unhealthy by fulfilling ≥1 out of 4 metabolic syndrome components. Definition C (empiric); Metabolically healthy by fulfilling 0 out of 3 components including waist-to-hip ratio, systolic blood pressure and diabetes, and metabolically unhealthy by fulfilling ≥1 of the 3 components. Abbreviations: MHO; Metabolically healthy obesity, MUO; Metabolically unhealthy obesity, BMI; Body mass index, HDL-C; High-density lipoprotein cholesterol. The values represent age-adjusted mean levels (95% confidence intervals). (DOCX) [file pone.0333402.s002.docx]

| S2 Table. Mean age-adjusted levels of anthropometric and metabolic characteristics in MHO and MUO women and men by three definitions of metabolic health. The Tromsø Study 2015-2016. | | | | | | | | | | | | |
| --- | --- | --- | --- | --- | --- | --- | --- | --- | --- | --- | --- | --- |
|  | **Women** | | | | | | **Men** | | | | | |
|  | **Definition A**  **(MetS)** | | **Definition B**  **(strict)** | | **Definition C (empiric)** | | **Definition A**  **(MetS)** | | **Definition B**  **(strict)** | | **Definition C (empiric)** | |
|  | MHO n=1 217 | MUO n=1 199 | MHO n=442 | MUO n=1 974 | MHO n=692 | MUO n=1 724 | MHO n=950 | MUO n=1 520 | MHO n=251 | MUO n=2 219 | MHO n=443 | MUO n=2 027 |
| HbA1c, % | 5.57 (5.53-5.62) | 6.05 (6.01-6.10) | 5.54 (5.47-5.61) | 5.87 (5.84-5.90) | 5.60 (5.55-5.66) | 5.89 (5.86-5.93) | 5.58 (5.54-5.63) | 6.10 (6.06-6.14) | 5.59 (5.49-5.68) | 5.94 (5.91-5.97) | 5.62 (5.55-5.69) | 5.96 (5.93-5.99) |
| Systolic blood pressure, mmHg | 127.4 (126.4-128.4) | 135.7 (134.6-136.7) | 116.9 (115.3-118.5) | 134.8 (134.0-135.5) | 116.7 (115.4-117.9) | 137.5 (136.8-138.3) | 133.4 (132.3-134.6) | 138.2 (137.3-139.1) | 120.8 (118.7-123.0) | 138.1 (137.4-138.8) | 120.8 (119.3-122.4) | 139.7 (139.0-140.4) |
| Diastolic blood pressure, mmHg | 73.0 (72.4-73.5) | 75.6 (75.1-76.2) | 68.9 (68.0-69.8) | 75.5 (75.1-75.9) | 69.0 (68.3-69.7) | 76.5 (76.0-76.9) | 79.1 (78.5-79.7) | 81.2 (80.7-81.7) | 72.7 (71.5-73.8) | 81.3 (80.9-81.7) | 73.2 (72.3-74.1) | 81.9 (81.5-82.3) |
| Triglycerides, mmol/l | 1.20 (1.17-1.23) | 1.96 (1.95-2.04) | 1.07 (1.02-1.12) | 1.68 (1.64-1.71) | 1.33 (1.28-1.38) | 1.64 (1.60-1.68) | 1.39 (1.35-1.43) | 2.25 (2.21-2.30) | 1.21 (1.14-1.28) | 1.97 (1.93-2.00) | 1.71 (1.64-1.79) | 1.91 (1.87-1.95) |
| HDL-C, mmol/l | 1.63 (1.61-1.65) | 1.31 (1.29-1.33) | 1.72 (1.68-1.75) | 1.42 (1.40-1.43) | 1.53 (1.51-1.56) | 1.45 (1.43-1.46) | 1.36 (1.34-1.38) | 1.11 (1.10-1.13) | 1.38 (1.34-1.42) | 1.19 (1.17-1.20) | 1.25 (1.22-1.29) | 1.20 (1.18-1.21) |
| Waist circumference, cm | 105.2 (104.6-105.7) | 108.9 (108.4-109.5) | 103.8 (102.9-104.7) | 107.8 (107.3-108.2) | 102.4 (101.7-103.1) | 108.9 (108.5-109.3) | 111.9 (111.3-112.5) | 114.4 (114.0-114.9) | 110.7 (109.5-111.8) | 113.8 (113.4-114.2) | 108.2 (107.3-109.1) | 114.6 (114.2-115.0) |
| Waist-to-hip ratio | 0.91 (0.91-0.91) | 0.94 (0.94-0.94) | 0.90 (0.90-0.91) | 0.93 (0.93-0.93) | 0.88 (0.88-0.89) | 0.94 (0.94-0.94) | 1.01 (1.00-1.01) | 1.03 (1.02-1.03) | 1.00 (0.99-1.00) | 1.02 (1.02-1.02) | 0.97 (0.97-0.98) | 1.03 (1.03-1.03) |
| BMI, kg/m^2^ | 33.5 (33.3-33.7) | 34.6 (34.4-34.8) | 32.9 (32.7-33.1) | 34.3 (34.2-35.4) | 33.0 (32.7-33.2) | 34.5 (34.3-34.6) | 32.7 (32.5-32.9) | 33.5 (33.3-33.6) | 32.4 (32.2-32.7) | 33.3 (33.2-33.3) | 31.7 (31.4-32.0) | 33.5 (33.3-33.6) |

Definition A (MetS); Metabolically healthy by fulfilling ≤1 out of 4 metabolic syndrome components, and metabolically unhealthy by fulfilling ≥2 out of 4 metabolic syndrome components. Definition B (strict); Metabolically healthy by fulfilling 0 out of 4 metabolic syndrome components, and metabolically unhealthy by fulfilling ≥1 out of 4 metabolic syndrome components. Definition C (empiric); Metabolically healthy by fulfilling 0 out of 3 components including waist-to-hip ratio, systolic blood pressure and diabetes, and metabolically unhealthy by fulfilling ≥1 of the 3 components. Abbreviations: MHO; Metabolically healthy obesity, MUO; Metabolically unhealthy obesity, BMI; Body mass index, HDL-C; High-density lipoprotein cholesterol. The values represent age-adjusted mean levels (95% confidence intervals).
