# Supplementary figures and images for: Assessing metabolic health in a general population: A comparative analysis of three definitions in the Tromsø Study 2015–2016
Source: PLoS One. 2025 Oct 6;20(10):e0333402. doi: 10.1371/journal.pone.0333402 (PMC12500164; doi:10.1371/journal.pone.0333402)

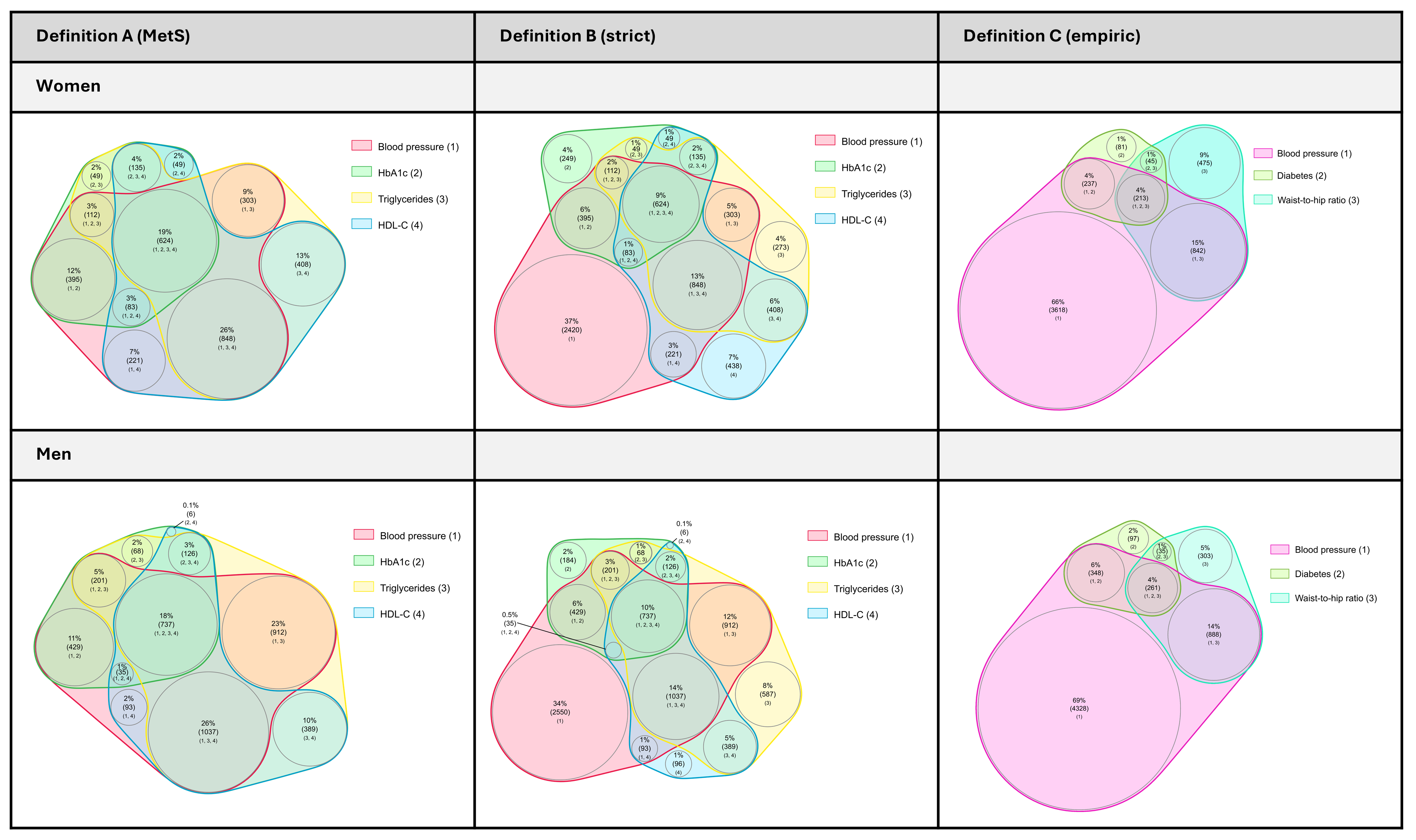

Supplement: S1 Fig — The Tromsø study 2015–2016. Definition A (MetS); Metabolically unhealthy by fulfilling ≥2 out of 4 metabolic syndrome components. Definition B (strict); Metabolically unhealthy by fulfilling ≥1 out of 4 metabolic syndrome components. Definition C (empiric); Metabolically unhealthy by fulfilling ≥1 out of 3 components including waist-to-hip ratio, systolic blood pressure and diabetes. Blood pressure (Definition A and B); Systolic blood pressure ≥130 mmHg and/or diastolic blood pressure ≥85 mmHg, and/or self-reported current use of blood pressure lowering drugs. HbA1c; HbA1c ≥6.0%, and/or self-reported diabetes, and/or self-reported current use of diabetes tablets or insulin. HDL-C; HDL-C <1.30 mmol/l (women) and <1.0 mmol/ (men), and/or self-reported current use of cholesterol-lowering drugs. Triglycerides; Non-fasting triglyceride levels ≥2.0 mmol/l, and/or self-reported current use of cholesterol-lowering drugs. Blood pressure (Definition C); Systolic blood pressure ≥130 mmHg, and/or self-reported current use of blood pressure lowering drugs. Diabetes; HbA1c ≥6.5%, and/or self-reported diabetes. Waist-to-hip ratio; Waist-to-hip ratio ≥0.95 (women), ≥1.03 (men). (TIF) [file pone.0333402.s003.tif]
